# Effect of Tensile Strain on Thermal Conductivity in Monolayer Graphene Nanoribbons: A Molecular Dynamics Study

**DOI:** 10.3390/s130709388

**Published:** 2013-07-22

**Authors:** Jianwei Zhang, Xiaodong He, Lin Yang, Guoqiang Wu, Jianjun Sha, Chengyu Hou, Cunlu Yin, Acheng Pan, Zhongzhou Li, Yubai Liu

**Affiliations:** 1 School of Electronic Science and Technology, Dalian University of Technology, Dalian 116024, China; E-Mails: jwzhang@dlut.edu.cn (J.Z.); ycl0913@mail.dlut.edu.cn (C.Y.); dlutcheng@mail.dlut.edu.cn (A.P.); lizhongzhou127@163.com (Z.L.); yubail88@gmail.com (Y.L.); 2 Centre for Composite Materials and Structures, Harbin Institute of Technology, Harbin 150080, China; E-Mail: Charlie@hit.edu.cn; 3 The state Key Laboratory of Structural Analysis for Industrial Equipment, Dalian University of Technology, Dalian 116024, China; E-Mails: gqwu@dlut.edu.cn (G.W.); jjsha@dlut.edu.cn (J.S.); 4 School of Electronics and Information Engineering, Harbin Institute of Technology, Harbin 150080, China; E-Mail: houcy@hit.edu.cn

**Keywords:** graphene nanoribbons, thermal conductivity, phonon

## Abstract

The thermal conductivity of monolayer graphene nanoribbons (GNRs) with different tensile strain is investigated by using a nonequilibrium molecular dynamics method. Significant increasing amplitude of the molecular thermal vibration, molecular potential energy vibration and thermal conductivity vibration of stretching GNRs were detected. Some 20%∼30% thermal conductivity decay is found in 9%∼15% tensile strain of GNR cases. It is explained by the fact that GNR structural ridges scatter some low-frequency phonons which pass in the direction perpendicular to the direction of GNR stretching which was indicated by a phonon density of state investigation.

## 1. Introduction

Graphene nanoribbons (GNRs) are several layers of two-dimensional honeycomb lattice of sp^2^ bonded carbon graphite. GNRs have high in-plane electron mobility and high out-plane electron resistance, making them the most promising candidates for the next generation of logic devices and sensors [1]. Potential uses of GNRs for different electronic devices, such as radio-frequency integrated circuits, thermal transistors, thermal rectifier, microscopic refrigerators and microelectronic processor coolers, have been conceptualized [2–7].

The thermal conductivity of GNRs plays an important role on the design and application of high quality GNR electronic devices. Investigations by experiments and molecular dynamic (MD) simulations have demonstrated that the thermal conductivity of graphene is ultrahigh [5,8,9]. Results found that the thermal conductivity of GNRs is associated with the chirality, layer and size of graphene, as well as other factors [10–13]. MD simulations show that the thermal conductivity of single-layered zigzag graphene is 20% higher than that of the single-layered armchair ones [5]. The thermal conductivity of graphene decays monotonically with increasing the number of the layers in few-layered graphene, which could be attributed to the cross-plane coupling of the low-energy phonons and changes in the phonon Umklapp scattering [11,14]. The MD simulations also indicated that the thermal conductivity of graphene reduces significantly with a little hydrogen coverage [15].

Stretching of GNRs would be a critical issue in the manufacture and application of GNR electronic devices, such as the graphene resonators for showing the images of scanning electron microscopy (SEM) [3,16]. Actually, the maximum tensile strain that monolayer GNR will experience is about 15% [12,17–19].

Therefore, an in depth investigation concerning the relationship between the tensile strain and thermal conductivity of GNRs is quite necessary. The measurement of GNR's thermal conductivity from experiments is quite difficult due to their limited size. In the present work, in order to correlate the thermal conductivity with the tensile strain of mono-layered GNRs, a non-equilibrium molecular dynamic method (NEMD) with quantum correction was applied to construct the MD models, and then the effect of tensile strain on the thermal conductivity of GNRs was investigated [20].

## 2. Model Construction for MD Simulation

For MD simulations, firstly, the classical MD method based on the COMPASS potential function is applied to describe the valence-bond and non-bond energy of graphene. The COMPASS force field calculation fits well with that of experiment work, and has been verified in simulating the mechanical properties of carbon nanotubes (CNTs) [21].

The COMPASS force field is first a high quality forcefield and focuses on high accuracy in prediction. It is an *ab initio* forcefield because most parameters were derived based on *ab initio* data. Compared with other force fields, COMPASS valence terms include high-order (cubic and quartic) bond, angle, torsion angle, out-of-plane angle terms, and the cross-coupling terms between them [22]. The COMPASS force field is suitable and has a high efficiency for simulating the thermal motion-caused molecular structural deformation and variations. The functions for non-bonding energy in COMPASS potential include the van der Waals (vdW) term and the Coulombic electrostatic term [22].

**Figure 1.**
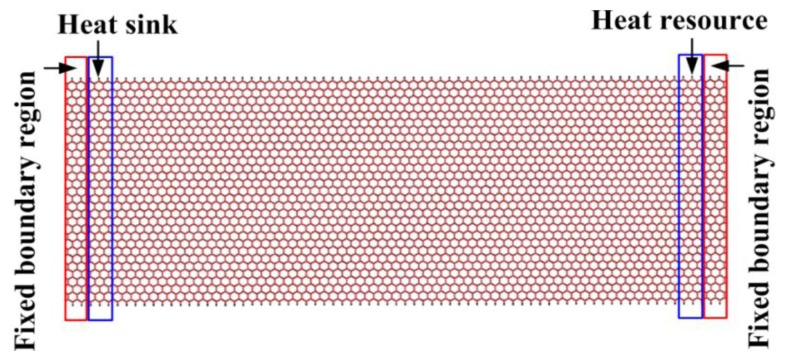
The MDs model for the simulation of thermal conductivity of GNRs.

Figure 1 shows the model for a GNR with a size of 16 nm and 6 nm in length and width, respectively. The size of the model in our work is relative large in comparison to that of the literature [8,9,14,15,23]. In order to avoid the contraction of the stretched GNRs, the boundary conditions constraining the displacement along the length direction were applied, as shown in Figure 1. Also, to make the thermal transfer along the desired temperature gradient imposed by the NEMD approach, a heat sink (≤0.5 nm) with a temperature of 300 K (T_sink_) was applied on the left. On the right, a heat source with a temperature of 500 K (T_resource_) was applied. Then the response as the resulting heat flux was measured.

To ensure a reasonable molecular configuration, before NEMD calculations, atoms are assumed to achieve equilibrium in a NVT Nosé–Hoover thermostat (300 K) for 10,000 time steps with a fixed time step of 1.0 fs [21]. GNR structures are then fully optimized by an energy minimization process.

After the equilibration, NEMD approach run for 3 × 10^5^ time steps giving a total molecular dynamics time is 0.3 ns which is a sufficiently time for a COMPASS force field model [21]. After the system reaches heat conduction equilibrium, the thermal conductivity (ê) of the GNRs was calculated according to Fourier's law:

$$\hat{\text{e}}=J(t)/(\ddot{A}TS),$$

where *J* is the heat flux, *ÄT* is the temperature gradient along the length direction, *S* is the cross-section area of graphene layers.

The cross-section area of GNR layers is a product of the width of GNF and the thickness of graphene layer. In order to verify the feasibility of our simulation approach, the thickness of single graphene layer was used as 0.335 nm referred to a previous simulation [14].

It was found the thermal conductivity in the strain-free GNR is 525 Wm^−1^·K^−1^, which is in agreement well with that of the previous study [5]. Furthermore, by calculating the temperature distribution along the length direction of GNR slices, an approximate linear temperature gradient along the desired heat flow channel was found. The temperature of each slice of the GNR was determined by the following relationship:

(1) $$\frac{3}{2}Nk_{B}T=\sum_{i=1}^{N}\frac{1}{2}m_{i}{\left(V_{i}^{T}\right)}^{2}$$

where N is the number of atoms in a particular slice of the GNR, $V_{i}^{T}$ is the atom's velocity, *m_i_* is the atom's mass, and *k_B_* is the Boltzmann constant.

## 3. Results and Discussion

**Figure 2.**
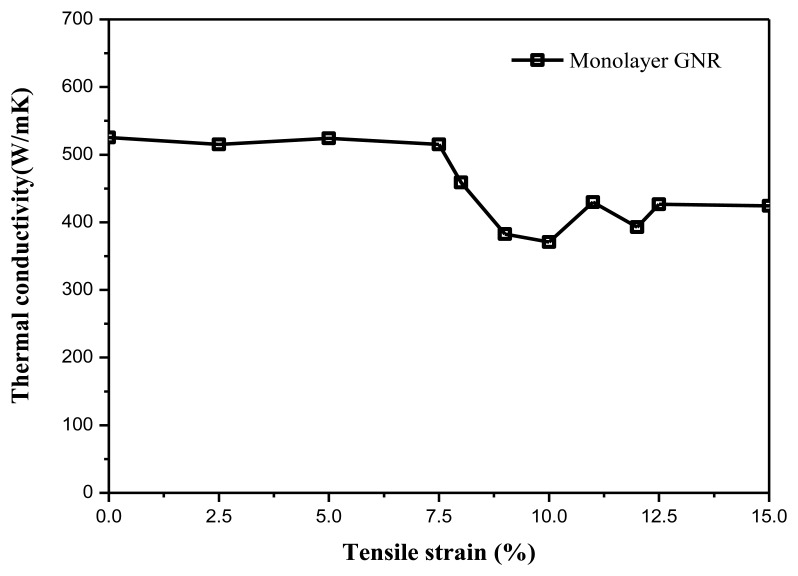
Tensile strain-dependent thermal conductivity of monolayer GNR.

For the train-free GNR, MD simulations show that there are some structural distortions on the graphene surface that look like ripples (in Figure 2a), that is graphene roughness, which seemed to be a necessary condition for the existence of single layer graphene [24]. The therrmal environment and heat conduction induce vibration on GNR atoms. This GNR atoms’ vibration engenders an undulation of the molecular potential energy described by the COMPASS force field. Graphene surface ripples also move up and down in this thermo-motion MD simulation. In some other MD simulation studies of graphene thermal conductivity by using other MD force fields, graphene atomic configurations do not have observable ripples. This means the COMPASS force field has higher accuracy in prediction of graphene structural variation in comparison with other force fields. The COMPASS force field is thus suitable for studying ripple formation triggered by tensile train.

To understand the effect of GNR tensile strain, MD simulations were performed for the same size and temperature gradient GNRs with different tensile strains, namely, 2.5%, 5%, 7.5%, 8%, 9%, 10%, 11%, 12%, 12.5%, 15%, separately. We find GNR under a larger tensile strain yields necking due to Poisson's contraction in the length direction, and this phenomenon is evident in close-up SEM images of suspended epitaxial graphene resonators [3,16].

MD simulation results show GNRs with different tensile strains have different thermal conductivities. In Figure 2, while the tensile strain varies from 0% to 7.5%, the thermal conductivity basically remained stable at about 525 Wm^−1^·K^−1^. Then the thermal conductivity begins to rapidly decrease to 371 Wm^−1^·K^−1^ while the tensile strain changes from 7.5% to 10.0%, which is almost 30% smaller than that of a strain-free GNR.

**Figure 3.**
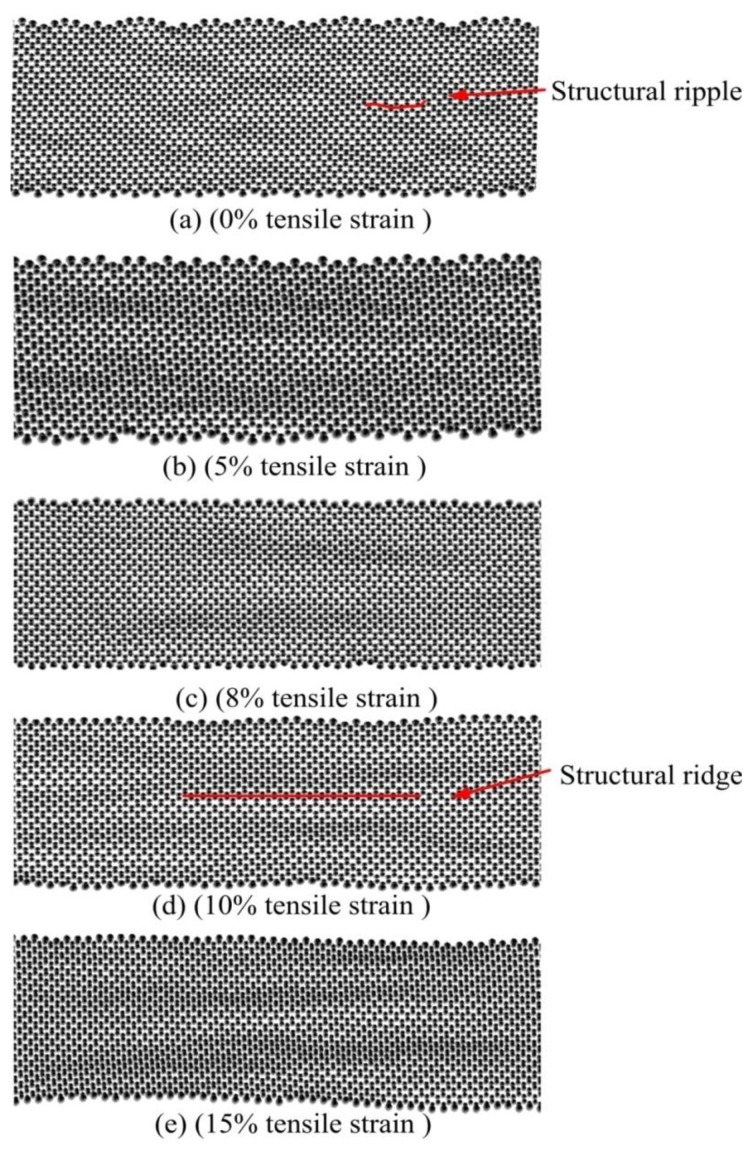
Graphene surface ripples change to ridges as the tensile strain increases: (**a**) 0% tensile strain; (**b**) 5% tensile strain; (**c**) 8% tensile strain; (**d**) 10% tensile strain; (**e**) 15% tensile strain.

The change of thermal conductivity relates to some molecular configuration changes of GNRs. Graphenes under different tensile strains exhibit different atomic configuration characteristics. In graphene under tensile strain bigger than 7.5% cases, graphene's structural ripples are clearly elongated and changed into some structural ridges which have larger sizes in the stretching direction. These structural ridges of stretching GNR (shown in Figures 3c,d,e) have also been observed by SEM [3,16]. Based on the investigation of atomic configurations variation in our simulations (Figure 3), we find the GNR's atoms’ thermal vibration increases dramatically when the tensile strain increases. By calculation of the molecular potential energy of GNRs, we observed how the tensile strain brings about increasing molecular potential energy vibrations of the stretching GNR. For example, in our simulations, the average amplitude of potential energy fluctuation per atom for strain-free graphene is about 1 Kcal/mole and for 10% tensile strain graphene is about 4 Kcal/mole. This may be due to the main body of thermal motion changing from ripples to larger sized ridges. The stretching GNR's thermal conductivity *versus* time curve also exhibits an outrageous oscillation caused by the increasing potential energy vibration. The final values of thermal conductivity were determined by averaging 400 fore-and-aft time step points in calculated thermal conductivity versus time step curve.

This thermal conductivity decrease coincides with the generation of GNR structural ridges. It implies a stretching graphene's thermal conduction is influenced by the structural ridges on the GNR. When the tensile strain increases from 12.5% to 15%, the thermal conductivity remains steady at about 425 Wm^−1^·K^−1^.

Theoretical and experimental studies have demonstrated two-dimensional phonon transport in nano-ribbons [5,23,25,26]. A large number of thermal phonons propagate across the GNR and pass though the structural ridges in the stretched GNRs [26]. We investigated phonon density of states of GNRs in this MD simulation study. In the cases where the tensile strain was larger than 7.5%, phonons passing perpendicular to the longitudinal direction of GNR are scattered in a lower frequency band (from 0 to 400 cm^−1^) compared with that of strain-free GNR. These phonons scattered by structural ridges result in a decrease in stretching GNR thermal conductivity.

The thermal conductivity reduction is attributed to the increasing lattice anharmonicity of stretching GNR [27]. Thermal conductivity of single-layer zigzag graphene is much larger than that of the single-layer armchair graphene [5]. It implies that the scattering of phonons passing and reflecting between GNR edges can seriously affect GNR's thermal conductivity likes this study suggests. Moreover, graphene's thermal conductivity is contributed by its bending mode [14]. Compared with the structural ripples, structural ridges increase the bending of the graphene plane and result in a thermal conductivity decrease.

## 4. Conclusions

In summary, molecular dynamic simulations have shown that GNR surface ripples will be changed into structural ridges by tensile strain. The tensile stretching of a GNR results in a significant increase of amplitude of the molecular thermal vibration and thermal conductivity vibration of the GNR. The thermal conductivity of GNR can be decrease 20%–30% by stretching. It is explained as that phonon waves propagating across graphene structural ridges will be scattered by these graphene structural ridges and cause a decrease in thermal conductivity. Stretching should be an effective way to control the thermal conductivity of monolayer nanostructures.
